# Biomechanical and Microbiological Analysis of Canine Cadavers Fixed With Ethyl Alcohol Formaldehyde Mixtures for Anatomy and Surgery Education

**DOI:** 10.1111/ahe.70040

**Published:** 2025-05-14

**Authors:** Sérgio S. Alves, Andréa B. P. S. Queiroz, Nathalia T. Brandão, Geovana C. Ferreira, Raphael C. Zero, Fabrício S. Oliveira

**Affiliations:** ^1^ Department of Animal Morphology and Physiology School of Agrarian and Veterinary Science, São Paulo State University (UNESP) Jaboticabal São Paulo Brazil

**Keywords:** anatomy, biomechanical analyses, microbiology, preservation, surgery

## Abstract

In Brazil, with the creation of the Arouca Law in 2009 and the need for substitutes for live animals in studies, it is essential to apply anatomical techniques to conserve corpses. Fixative substances prevent autolysis, facilitate incisions and make the protein fraction of the tissue insoluble, preserving its morphology due to antiseptic properties. Preservative solutions aim to maintain anatomical specimens intact to allow the long‐lasting use of them. Several techniques can promote such fixation and preservation, but formaldehyde is the most used in many countries. This research aims to determine the viability of a new anatomical technique using ethyl alcohol (EA) and formaldehyde, in different proportions, to fix canine cadavers and sodium chloride aqueous solution (SCAS 30%) for preservation biomechanical and microbiological analyses. Fresh samples were collected before fixation to be the control samples in every group. Corpses were divided into four groups: G1 (only formaldehyde), G2 (30% formaldehyde and 70% EA), G3 (70% formaldehyde and 30% EA) and G4 (50% formaldehyde and 50% EA) and were subsequently conserved in 30% SCAS. Analyses were done at D0 (before fixation), D30, D60, D90 and D120 after preservation on 30% SCAS. Biomechanical traction tests were performed on skin and jejunum samples at all times of fixation and preservation. Microbiological analyses of the solution were at the end of fixation and during all preservation moments. The control samples (fresh corpses) were compared to the other four groups with the T‐test. There was no statistical difference in the maximum rupture force (MRF) of the skin and jejunum between the control and the fixation and preservation moments. It was observed that G2 and G3 presented minor variations in the MRF with means of skin (−14.2 N) and jejunum (−0.28 N). There were significant differences at all times for rupture elongation (RE) of the skin and jejunum. G3 and G4 showed minor variations in the RE, with a difference between the skin (1.32 mm) and jejunum (0.23 mm). The microbiological analyses of the SCAS 30% did not show any contamination (aerobic and anaerobic microorganisms) for Groups 1, 2 and 3. For D120 of G4, *Bacillus* spp. was identified in the amount of 1.0 × 10.

## 1 Introduction

The fixative solution prevents autolysis, facilitates tissue cutting and makes the protein fraction insoluble, preserving structural components through antiseptic properties. Preservative solutions aim to keep the morphological structures intact to allow the long‐lasting use of the anatomical species (Campos et al. 2016).

Laboratory practice allows the study of the macroscopic anatomy of a cadaver, which is one of the pillars for graduation in veterinary medicine. Embalmed anatomical specimens need to provide learning equivalent to that of a fresh cadaver (Lombardero et al. 2017; Nam et al. 2020).

A saturated sodium chloride solution (SCAS) is used to preserve corpses (Oliveira 2014; Lombardero et al. 2017). Cadavers preserved with this solution maintain more realistic colour, texture, joint movement and flexibility than those preserved in formaldehyde (Nam et al. 2020; Beger et al. 2020).

Recent studies present biomechanical analyses of tissues of dogs and cats during preservation (Cerqueira et al. 2017; Pelogia et al. 2018; Rocha et al. 2018; Fração et al. 2019; Zero et al. 2020; Queiroz, Rodrigues, Cardozo, Costa, Soares, et al. 2022; Queiroz et al. 2023), as well as microbiological analyses of fixative/conservative solutions (Pereira et al. 2019; Queiroz, Rodrigues, Cardozo, Costa, Soares, et al. 2022; Queiroz et al. 2022, 2023) and evaluation of students on surgical practice (Rocha et al. 2019; del Ponti et al. 2021). Formaldehyde‐free solutions are also being used for embalming dogs for cerebrospinal fluid collection training (Zero et al. 2022) and to perform thoracic procedures or dissections (Kihara et al. 2024).

This study aims to determine the feasibility of a new anatomical technique using pickling salt, ethyl alcohol and 10% formaldehyde in different proportions in the preservation of canine cadavers by biomechanical and microbiological analysis throughout the process.

## 2 Materials and Method

In the study, 32 canine cadavers ( *Canis familiaris* ) were used, 13 males and 19 females, adults, who died from causes that did not involve evident morphological changes, such as large tumour masses, extensive lacerating trauma or bone fractures. All came from the Zoonosis Control Center of Ribeirão Preto, SP, after approval by the Municipal Legal Department (process 02.2014/000027–1) and already have approval from the ethics committee of Unesp Campus de Jaboticabal for use (process 4593/19). Immediately after death, the animals were frozen (freezer at −18°C) for a period of 2 months and transported to the Animal Anatomy Laboratory at Unesp in Jaboticabal, SP, located 50 km away.

Animals weighed between 5 and 15 kg and had a body condition score of 4 (ribs easily palpable, with minimal fat coverage; abdominal girdle easily noticeable when viewed dorsally; abdominal fold evident) or 5 (ribs palpable and without excess fat coverage; abdominal waist observed caudal to the ribs when viewed dorsally; abdominal fold evident when viewed laterally), considered ideal by Laflamme (1997), on a scale of 1 to 9, were selected in the present experiment. These cadavers were selected post‐mortem once they were already located in the freezer of the surgical anatomy laboratory (LAC).

All animals received individual identification, using a plastic film marked with a pyrograph, referring to the group, number and weight, and were fixed to the thoracic limb with a string.

## 3 Anatomical Technique

The animals were thawed in water at room temperature (between 20°C and 25°C) for 12 h inside a water tank, totally shaved and randomly distributed into four groups of eight animals, in which the picking salt solution was infused followed by the fixative solution composed of ethyl alcohol (EA) and 10% formaldehyde (10% F) in which the 10% formaldehyde solution was prepared by diluting 100 mL of 40% pure formaldehyde with 900 mL of water, in different proportions (Table 1).

**TABLE 1:** Mean and standard deviation of body weight of canine cadavers and anatomical techniques used by the group (G1: 100% formaldehyde, G2: 70% ethyl alcohol and 30% formaldehyde; G3: 30% ethyl alcohol and 70% formaldehyde; G4: 50% ethyl alcohol and 50% formaldehyde), after infusion of Pickling salt solution.

Group	Number of cadavers	Weight (Kg)	Anatomical technique	
			Ethyl alcohol (EA)	10% formaldehyde (10% F)
G1	8	8.33 ± 2.35	—	100%
G2	8	7.94 ± 2.04	70%	30%
G3	8	8.29 ± 2.14	30%	70%
G4	8	8.91 ± 3.22	50%	50%

**FIGURE 1:**
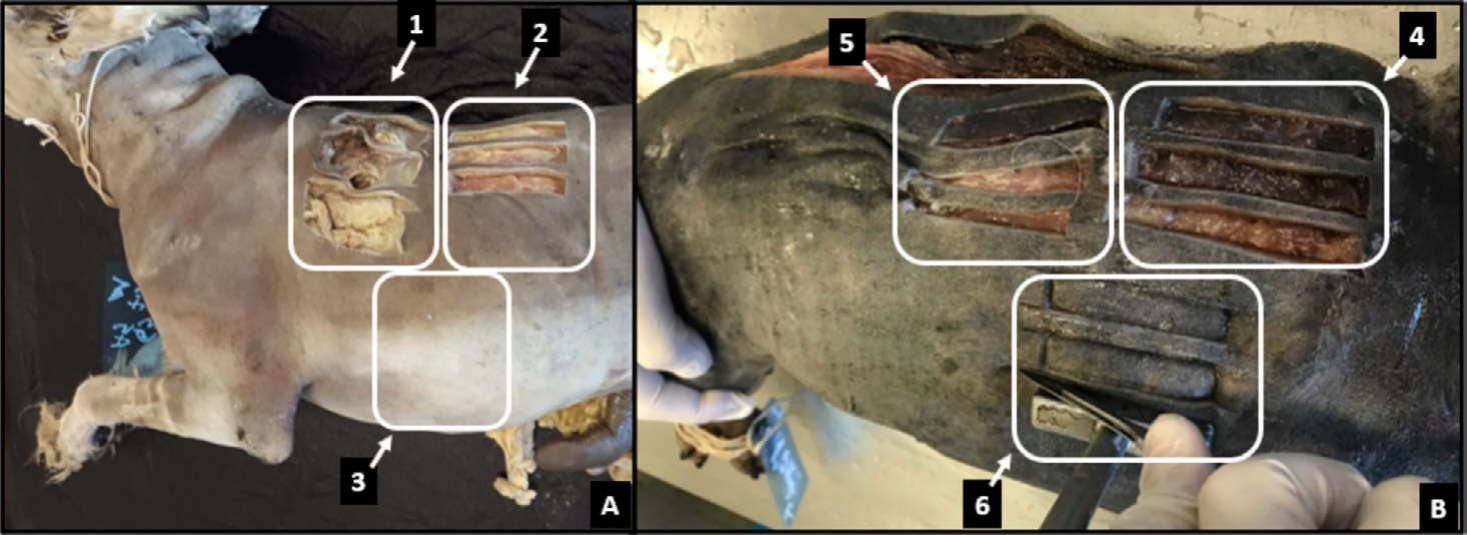
Chemically preserved dog skin collection sites. (A) Left antimere and collection of samples in triplicate at fresh times (control sample), D0 (considering the 7 days that the cadavers were immersed in a 10% formaldehyde solution plus 2 (two) days of washing before placing them in the tank with SCAS 30%) and D30 (30 days of preservation in SCAS 30%) arrows with numbers 1, 2 and 3 respectively; (B) Right antimere and sample collection in triplicate at moments D60 (60 days of preservation in SCAS 30%), D90 (90 days of preservation in SCAS 30%) and D120 (120 days of preservation in SCAS 30%) arrows with numbers 4, 5 and 6 respectively.

A solution with pickling salt (PSS) containing 20% NaCl + 1% nitrite + 1% sodium nitrate was injected via the common carotid artery (120 mL/kg), where this artery was dissected and cannulated (Figure 1) and then infused as a fixative, a solution composed of ethyl alcohol and formaldehyde in different proportions (150 mL/kg) in each group, as described below:

− Group 1 (G1): formaldehyde (10%) as a fixative solution.
− Group 2 (G2): 30% formaldehyde (10%) and 70% EA.
− Group 3 (G3): 70% formaldehyde (10%) and 30% EA.
− Group 4 (G4): 50% formaldehyde (10%) and 50% EA.

All groups were evaluated before fixation and at 0, 30, 60, 90 and 120 days of preservation in 30% SCAS.

After the injection of the fixative, an incision of approximately 14 cm was made in the midline of the abdomen to remove the accumulated fluid using water. The descending colon and rectum region were cleaned with a 0.25% sodium hypochlorite solution to remove excreta using a 20 cm long urethral probe coupled to a 60 mL syringe. This procedure was important to reduce contamination of the preservation solution in which the cadavers remained throughout the experiment.

After this procedure, the cadavers were put in 310 L of plastic boxes (1 box per group) and a screw‐on lid containing 180 L of 10% formaldehyde and remained for 7 days to complete the process of fixation. Afterwards, the corpses were washed in tap water for 2 days and then placed in boxes with 180 L of 30% SCAS for preservation.

## 4 Material Collection

A 1 × 5 cm stainless steel mould was used, and three skin and jejunum samples were collected before the infusion of the fixation and preservation solutions, which were immediately subjected to the biomechanical traction test. The fresh samples served as controls, and after this procedure, these cadavers were subjected to fixation and subsequently put in plastic boxes with the preservation solution.

To skin samples, the cadaver was positioned in the right lateral decubitus position. With the help of a scalpel (blade number 24), the mould was contoured by excising the cutaneous fragments of skin collected longitudinally (craniocaudal), which is the direction that provides greater traction resistance, as demonstrated in sheep (Jacinto et al. 2004; Haar et al. 2013). For the fresh samples (control samples), D0 and D30, the collection was carried out on the left antimere and at D60, D90 and D120, on the right antimere in all groups (Figure 1).

For the jejunum collection, animals were positioned in dorsal decubitus, and the jejunum was externalised by manual traction through a median celiotomy. After identifying the duodenojejunal flexure, the steel mould was positioned over the intestine, delimiting with Metzenbaum scissors in a longitudinal direction. Subsequently, the mesenteric surface was sectioned, exposing the lumen. The steel mould was placed, and the three samples were taken out. Immediately after collecting, the skin and jejunum samples were placed in individually identified bottles containing water and immediately transported to the Laboratory of Surgical Anatomy, Department of Animal Morphology and Physiology at FCAV—UNESP, Jaboticabal Campus, for biomechanical analyses.

## 5 Analysis Regarding Tissue Resistance

To evaluate tissue resistance, a Universal Testing Machine (Model Emic DL 2000) was used, with a 500 N load cell and electromechanical drive support, with a speed of 100 mm/min. Manual compression drive traction claws were also used. The equipment belongs to the Department of Animal Morphology and Physiology at FCAV–UNESP–Jaboticabal.

## 6 Microbiological Analyses of Total Aerobic and Anaerobic Mesophilic Bacteria

The samples were collected at the end of fixation and during preservation, except on D0, that is, after 7 days in formaldehyde 10% and at times 30, 60, 90 and 120 days in 30% SCAS. A 100 mL bottle with a previously sterilised lid, identified as to the fixation/preservation medium, date, group and moment, was analysed. After collection, the material was duly sent to the Microbiology Laboratory at FCAV‐UNESP Jaboticabal to carry out microbiological analyses.

The quantification of viable aerobic mesophilic and facultative anaerobic bacteria was carried out using the deep plating technique, also known as Pour Plate. In this technique, the sample was diluted up to five times, and for each dilution, 1.0 mL was used as inoculum, in duplicate, distributed in previously sterilised Petri plates. Subsequently, the melted medium is poured and cooled to 45°C in a water bath onto the Petri plate containing the diluted sample suspension. The material was homogenised using circular movements clockwise and counterclockwise, repeated about 10 times. After the medium solidified, the plates were incubated in ovens at the appropriate temperature and atmosphere for 24 h.

To this end, the plates for counting aerobic microorganisms were stored directly in a bacteriological oven, while the plates for counting anaerobic microorganisms were stored in anaerobic jars using Aneroback (Probac) at 37°C for 24 h. The Colony Forming Unit (CFU) was counted using a magnifying glass.

After counting, five colonies were isolated from each plate, with different phenotypic characteristics among them, on BHI agar (Brain Heart Infusion) and then incubated at 37°C for 24 h for subsequent identification.

## 7 Bacterial Identification

The colonies were subjected to Gram staining for initial classification and then seeded in selective culture media for the genera analysed, namely: Mac Conkey Agar, for evaluation of *Pseudomonas* and *Escherichia coli* ; Polymyxin Sulfadizine Sulfate Agar (SPS), to identify the genus *Clostridium*; Mannitol Yolk Polymixin Base Agar (MYP), for isolation of *Bacillus*; and Azide Blood Agar for identification of *Staphylococcus*.

After incubation at 37°C for 24 h, the colonies were evaluated using the Gram method for cell morphology and were subsequently subjected to specific biochemical tests to confirm each genus or species (Barrow and Feltham 1993).

## 8 Muscle Colour Analysis

After the preservation period in 30% SCAS, incisions were made in the *M*. *biceps femoris, M*. *vastus lateralis*, *M*. *gluteus superficialis, M*. *gluteus medius* and *deltoideus (pars scapularis)*, *M. triceps brachii* (*caput longum* and *caput laterale*) and *m. triceps brachii* (*caput laterale*) assigning scores 1 at 5. The evaluation of the scores was always carried out by the same observer, a veterinarian with experience in preparing anatomical material (Table 2).

**TABLE 2:** Score used to identify the colour of the muscles after preservation.

Score	Colour
1	Yellow
2	Light red
3	Bright red
4	Dark red
5	Grey

## 9 Statistical Analysis

The Shapiro–Wilk test was used to verify whether the variables followed a normal distribution, and the homogeneity of variances was verified by the Brown‐Forsythe test. One‐class analysis of variance (One‐Way ANOVA) was used for repeated measures (TIMES) for each treatment separately. Also, for each treatment, a comparison was made between control samples and samples from groups obtained at later times using the paired T‐test. When necessary, non‐parametric tests were used (Friedman for repeated measures and Wilcoxon for paired data). In order to compare treatments, double classification analysis of variance for repeated measures (Two‐Way ANOVA) was used. The significance level adopted was 5%, and the statistical package used in the analysis was SigmaPlot 12.0.

## 10 Results

The application of the anatomical technique using the PSS followed by fixation with EA and 10% F in different proportions, and subsequently preservation in 30% SCAS, proved to be effective for fixing and preserving the dogs throughout the experiment. After the period of preservation in 10% formaldehyde for 7 days, as well as after the period of preservation in 30% SCAS, there was little release of fat from the cadavers in the solution present in the plastic boxes. Greater malleability of the cadavers to handling was observed after preservation in 30% SCAS, both concerning the thoracic and pelvic limbs, as well as the samples of skin and jejunum collected. In groups 2 and 3, there was greater release of fat, and this decreased over time at 30% SCAS. The samples were always easy to handle and collect.

Due to the use of PSS, the maintenance of the reddish colour of the muscles was observed, leaving them close to their real appearance (fresh corpse) and avoiding the greyish appearance of the muscles after formaldehyde injection, as well as the yellowish appearance when a greater amount of ethyl alcohol is used for fixing/preserving.

The *p* values of the treatments referring to maximum rupture force (MRF) and to the rupture elongation (RE) are shown in the tables below at a significance level of 5% by One‐Way ANOVA analysis of variance (Tables 3, 4, 5, 6). In Table 3, even though there is no statistically significant difference, it can be noted that the difference between the averages in maximum skin‐breaking force, referring to group 2, over a period of 120 days, was shown with numerical data closer to the control, with a value of −14.2 N. In Table 4, in the same way as Table 3, even though there was no statistically significant difference, it can be noted that the difference between the averages in maximum jejunal rupture force, referring to group 3, over a period of 120 days, was shown with numerical data closer to the control, with a value of −0.28 N. Regarding the maximum elongation at break, in Tables 5 and 6, there were statistical differences between the groups. However, it is worth mentioning that in Table 5, it can be noted that the difference between the averages in the maximum elongation at the break of the skin, referring to group 3, at a time of 120 days, was shown with numerical data closer to the control, with the value being 1.32 mm. In Table 6, it can be seen that the difference between the averages in the maximum elongation of jejunum rupture, referring to group 4, over a period of 120 days, was shown with numerical data closer to the control, with a value of 0.23 mm. Based on the present result, group 3 (30% ethyl alcohol and 70% formaldehyde) showed numerical values closer to the control, both in maximum breaking strength and maximum breaking elongation.

**TABLE 3:** Mean and standard deviation of the maximum rupture force (*N*) of the skin of canine cadavers chemically prepared using a pickling salt solution followed by fixation with ethyl alcohol and formaldehyde in different proportions and kept in 30% SCAS for up to 120 days.

Group	Parameters	Moments					
		Control	D0	D30	D60	D90	D120
G1	MRF (*N*)	95.78	89.73a	90.88a	82.36a	73.16b	70.84a
	Standard deviation	48.35	49.01	—	39.37	—	—
	Difference between means	—	6.05	4.9	13.42	22.62	24.94
	*p*	—	0.432	0.742	0.052	0.008	0.195
G2	MRF (*N*)	143.97	162.98a	174.88a	148.52a	144.78a	158.17a
	Standard deviation	—	—	—	—	—	92.76
	Difference between means	—	−19.01	−30.91	−4.55	−0.81	−14.2
	*p*	—	0.383	0.742	0.547	0.844	0.82
G3	MRF (*N*)	156.7	145.52a	162.73a	145.46a	135.01a	125.23a
	Standard deviation	73.55	60.03	74.17	64.52	67.5	36.73
	Difference between means	—	11.18	−6.03	11.24	21.69	31.47
	*p*	—	0.501	0.773	0.309	0.279	0.12
G4	MRF (*N*)	167.3	149.91a	119.62a	142.18a	114.53a	138.41a
	Standard deviation	123.61	130.72	—	93.35	—	81.62
	Difference between means	—	17.39	47.68	25.12	52.77	28.89
	*p*	—	0.6	0.148	0.197	0.383	0.352

*Note:* Moments: Control (fresh samples), D0 (considering the 7 days that the cadavers were immersed in a 10% formaldehyde solution plus 2 days of washing in running water), D30 (30 days), D60 (60 days), D90 (90 days) and D120 (120 days) in SCAS 30%. Means followed by different letters in the same line differ from each other at a significance level of 5% by One‐Way ANOVA analysis of variance.

**TABLE 4:** Mean and standard deviation of maximum rupture force (*N*) of the jejunum of canine cadavers chemically prepared using pickling salt solution followed by fixation with ethyl alcohol and formaldehyde in different proportions and kept in 30% SCAS for up to 120 days.

Group	Parameters	Moments					
		Control	D0	D30	D60	D90	D120
G1	MRF (*N*)	11.33	11.96a	18.51b	16.28a	15.69a	14.13a
	Standard deviation	5.33	8.27	5.89	6.51	9.2	4.7
	Difference between means	—	−0.63	−7.18	−4.95	−4.36	−2.8
	Valour de P	—	0.631	0.026	0.059	0.112	0.154
G2	MRF (*N*)	17.69	13.14a	16.73a	19.66a	15.73a	15.63a
	Standard deviation	8.66	6.79	5.87		5.45	9
	Difference between means	—	4.55	0.96	−1.97	1.96	2.06
	*p*	—	0.103	0.747	0.25	0.561	0.345
G3	MRF (*N*)	18.48	14.61a	19.59a	20.3a	18.25a	18.76a
	Standard deviation	5.48	9.01	9.51	7.98	3.27	6.18
	Difference between means	—	3.87	−1.11	−1.82	0.23	−0.28
	*p*	—	0.304	0.712	0.45	0.874	0.894
G4	MRF (*N*)	14.92	12.71a	13.04a	14.48a	15.12a	17.03a
	Standard deviation	7.86	8.63	5.15	6	6.34	7.91
	Difference between means	—	2.21	1.88	0.44	−0.2	−2.11
	*p*	—	0.382	0.585	0.895	0.958	0.637

*Note:* Moments: Control (fresh samples), D0 (considering the 7 days that the cadavers were immersed in a 10% formaldehyde solution plus 2 days of washing in running water), D30 (30 days), D60 (60 days), D90 (90 days) and D120 (120 days) in 30% SCAS. Means followed by different letters in the same line differ from each other at a significance level of 5% by One‐Way ANOVA analysis of variance.

**TABLE 5:** Mean and standard deviation of the rupture elongation (mm) of the skin of canine cadavers chemically prepared using a curing salt solution followed by fixation with ethyl alcohol and formaldehyde in different proportions and kept in 30% SCAS for up to 120 days.

Group	Parameters	Moments					
		Control	D0	D30	D60	D90	D120
G1	RE (mm)	7.47	7.42a	6.57a	7.7a	5.64b	5.38b
	Standard deviation	1.12	1.06	0.94	0.8	0.84	1.09
	Difference between means	—	0.05	0.9	−0.23	1.83	2.09
	*p*	—	0.917	0.094	0.527	0.005	< 0.001
G2	RE (mm)	8.81	8.73a	7.43b	7.8a	5.41b	5.57b
	Standard deviation	1	1.62	1.13	—	1.19	0.97
	Difference between means	—	0.08	1.38	1.01	3.4	3.24
	*p*	—	0.882	0.013	0.25	< 0.001	< 0.001
G3	RE (mm)	8.41	8.04a	7.17b	7.48a	5.23b	7.09a
	Standard deviation	0.88	1.16	—	1.81	0.63	2.03
	Difference between means	—	0.37	1.24	0.93	3.18	1.32
	*p*	—	0.458	0.008	0.499	< 0.001	0.148
G4	RE (mm)	7.79	7.33a	6.55a	7.11a	5.6b	6.03b
	Standard deviation	2.06	1.51	0.82	1.72	0.84	1.14
	Difference between means	—	0.46	1.24	0.68	2.19	1.76
	*p*	—	0.463	0.078	0.205	0.004	0.005

*Note:* Moments: Control (fresh samples), D0 (considering the 7 days that the cadavers were immersed in a 10% formaldehyde solution plus 2 days of washing in running water), D30 (30 days), D60 (60 days), D90 (90 days) and D120 (120 days) in 30% SCAS. Means followed by the same letter on the same line do not differ by paired *t*‐test.

**TABLE 6:** Mean and standard deviation of the rupture elongation (mm) of the jejunum of canine cadavers chemically prepared using a curing salt solution followed by fixation with ethyl alcohol and formaldehyde in different proportions and kept in 30% SCAS for up to 120 days.

Group	Parameters	Moments					
		Control	D0	D30	D60	D90	D120
G1	RE (mm)	6.92	6.99a	9.22a	8.14a	6.61a	9.31b
	Standard deviation	2.27	3.62	3.85	3.84	2.09	2.77
	Difference between means	—	−0.07	−2.3	−1.22	0.31	−2.39
	Valour de P	—	0.962	0.113	0.336	0.628	0.004
G2	RE (mm)	6.3	5.41a	6.71a	8.06a	7.89a	4.74b
	Standard deviation	1.89	0.88	3.99	4.08	3.43	1.49
	Difference between means	—	0.89	−0.41	−1.76	−1.59	1.56
	Valour de P	—	0.164	0.67	0.086	0.177	0.042
G3	RE (mm)	6.47	5.95a	7.76a	6.61a	6.51a	6.87a
	Standard deviation	3	3.31	3.42	2.15	2.84	3.06
	Difference between means	—	0.52	−1.29	−0.14	−0.04	−0.4
	Valour de P	—	0.691	0.15	0.863	0.97	0.687
G4	RE (mm)	7	6.87a	6.95a	8.16a	7.67a	6.77a
	Standard deviation	2.24	4.19	3.86	4.22	3.48	2.83
	Difference between means	—	0.13	0.05	−1.16	−0.67	0.23
	Valour de P	—	0.906	0.958	0.312	0.456	0.518

*Note:* Moments: Control (fresh samples), D0 (considering the 7 days that the cadavers were immersed in a 10% formaldehyde solution plus 2 days of washing in running water), D30 (30 days), D60 (60 days), D90 (90 days) and D120 (120 days) in 30% SCAS. Means followed by the same letter on the same line do not differ by paired *t*‐test.

**FIGURE 2:**
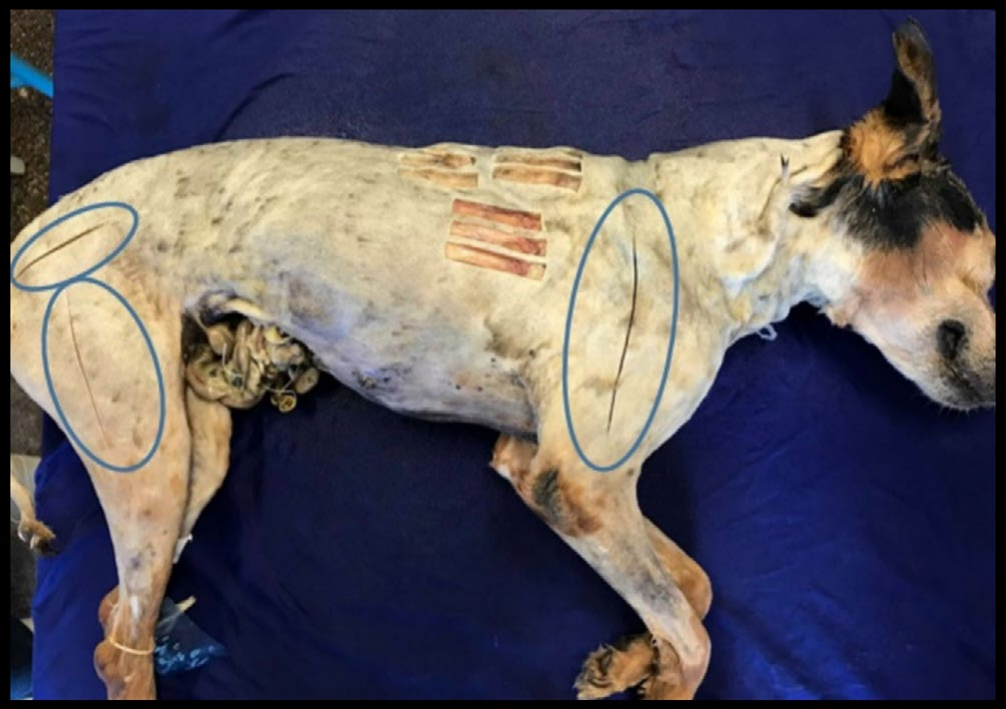
Locations where incisions were made for visual assessment of muscle colour in the different groups studied.

**FIGURE 3:**
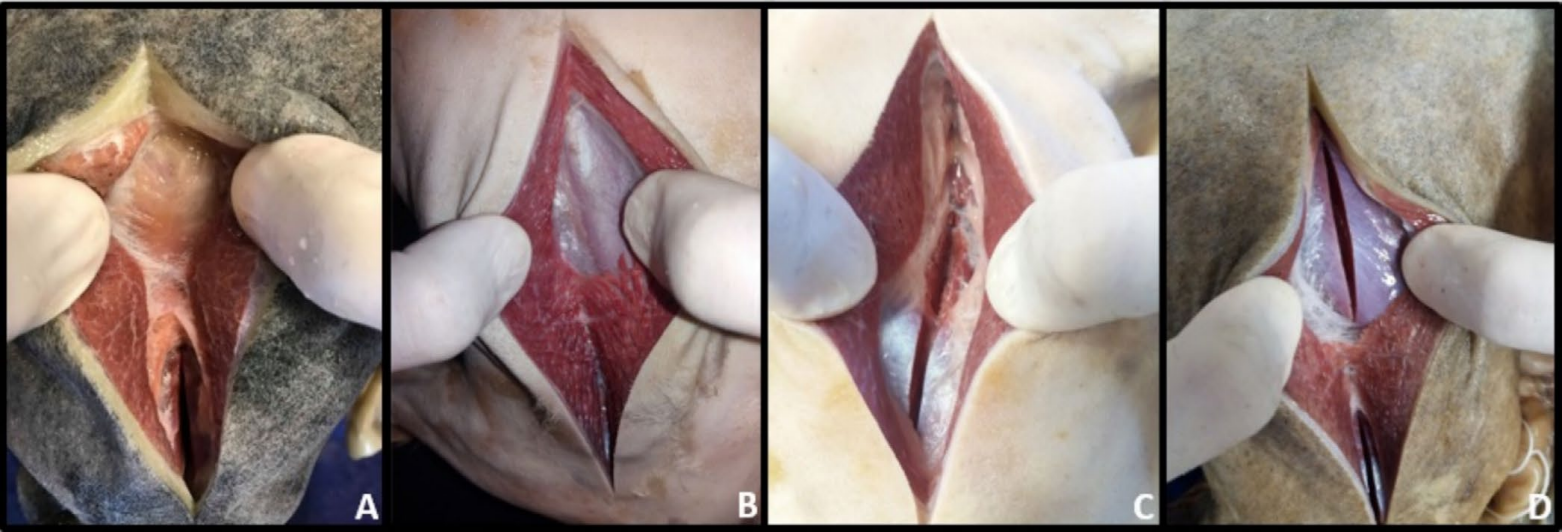
Muscular aspect of the pelvic limb region through section of the biceps femoris and vastus lateralis muscles after 120 days of preservation in 30% sodium chloride aqueous solution (SCAS 30%) being A (fixation with 100% formaldehyde), B (fixation with 70% ethyl alcohol and 30% formaldehyde), C (fixation with 30% ethyl alcohol and 70% formaldehyde) and D (fixation with 50% ethyl alcohol and 50% formaldehyde). All groups were infused with a curing salt‐based medium prior to the fixing agent. Note the light red colour in all groups, being more prominent in image B.

**FIGURE 4:**
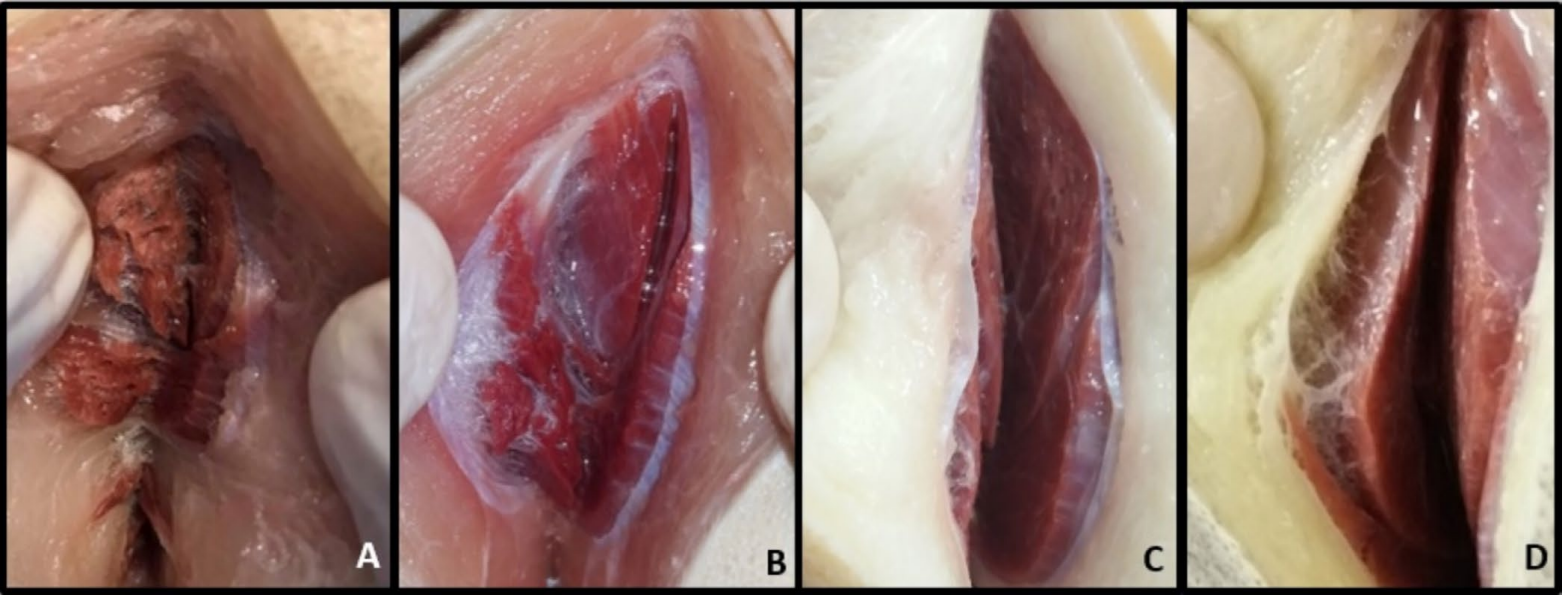
Muscle aspect of the gluteal region through a section of the gluteus superficialis and gluteus medius muscles after 120 days of preservation in 30% aqueous sodium chloride solution (SCAS 30%) being A (fixation with 100% formaldehyde), B (fixation with 70% ethyl alcohol and 30% formaldehyde), C (fixation with 30% ethyl alcohol and 70% formaldehyde) and D (fixation with 50% ethyl alcohol and 50% formaldehyde). All groups were infused with a curing salt‐based medium prior to the fixing agent. Note the light red colour in all groups, being more prominent in image B.

**FIGURE 5:**
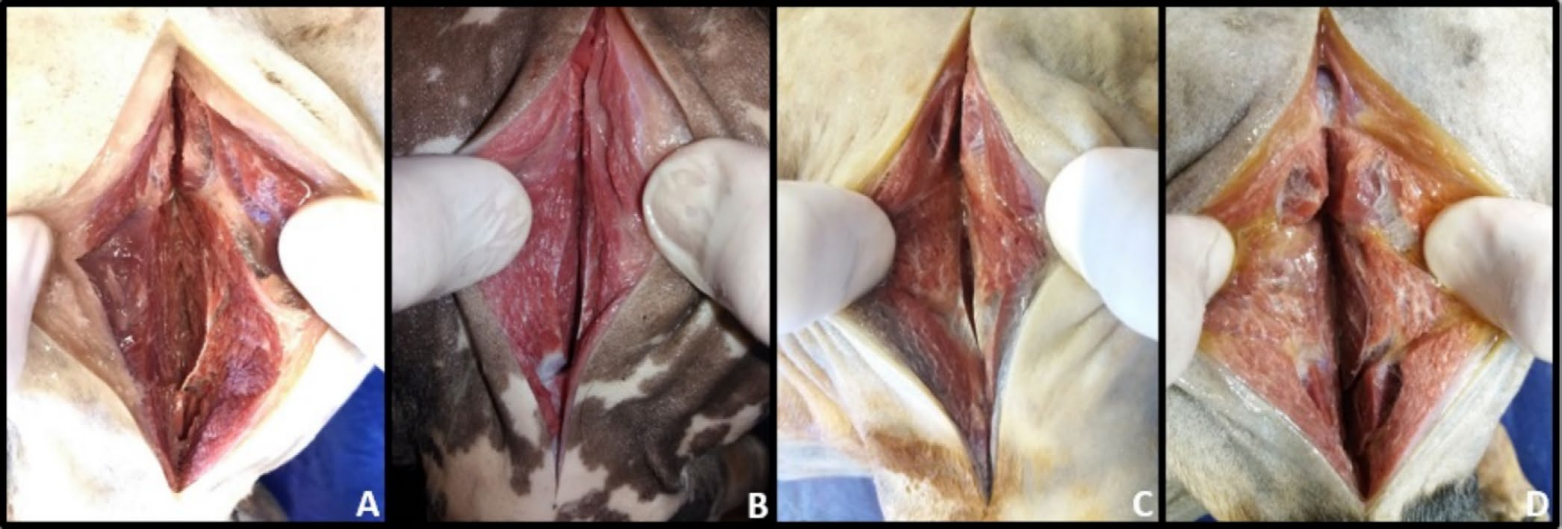
Muscular aspect of the arm region through section of the deltoid muscles, scapular portion, after 120 days of preservation in 30% aqueous sodium chloride solution (SCAS 30%) being A (fixation with 100% formaldehyde), B (fixation with 70% ethyl alcohol and 30% formaldehyde), C (fixation with 30% ethyl alcohol and 70% formaldehyde) and D (fixation with 50% ethyl alcohol and 50% formaldehyde). All groups were infused with pickling salt solution prior to the fixative agent. Note the light red colour in all groups, being more prominent in image B.

After 120 days of preservation in the different Groups, G1, G2, G3 and G4, incisions were made in the muscles for colour observation (Figures 2, 3, 4, 5 and Table 7).

**TABLE 7:** Score attributed to the colour of the muscles in the thoracic and pelvic limb regions after 120 days of preservation in SCAS 30%.

Muscular region	Groups	Score 1 Yellowish	Score 2 Light red	Score 3 Redish	Escore 4 Dark red	Escore 5 Grey
Pelvic limb region	Group 1%–100% F				X	
	Group 2%–30% F and 70% EA			X		
	Group 3%–70% F and 30% EA			X		
	Group 4%–50% F and 50% EA		X			
Gluteal region	Group 1%–100% F				X	
	Group 2%–30% F and 70% EA			X		
	Group 3%–70% F and 30% EA		X			
	Group 4%–50% F and 50% EA		X			
Thoracic limb region	Group 1%–100% F				X	
	Group 2%–30% F and 70% EA			X		
	Group 3%–70% F and 30% EA		X			
	Group 4%–50% F and 50% EA	X				

The microbiological analysis of the preservation solution for canine cadavers stored in 30% SCAS for Group 1, Group 2, Group 3 and Group 4, as well as during the storage period in the tank with 10% formaldehyde after 7 (seven) days and subsequently at times D30, D60 and D90, identified no aerobic and anaerobic microorganisms (or bacteria) Regarding the D120 time for group 4, *Bacillus* spp. aerobes were present in an amount of less than 1.0 × 10, as shown in Table 8.

**TABLE 8:** Microbiological analysis of the preservation solution of canine cadavers chemically prepared using a pickling salt solution followed by fixation with ethyl alcohol and formaldehyde in different proportions and kept in fixation with formaldehyde for 7 days and in 30% SCAS for up to 120 days.

	Groups	Total aerobics (CFU/mL)	Total anaerobics (CFU/mL)	Microorganisms
Formaldehyde‐based fixation medium	G1	0	0	Absent
	G2	0	0	Absent
	G3	0	0	Absent
	G4	0	0	Absent
D 30	G1	0	0	Absent
	G2	0	0	Absent
	G3	0	0	Absent
	G4	0	0	Absent
D 60	G1	0	0	Absent
	G2	0	0	Absent
	G3	0	0	Absent
	G4	0	0	Absent
D90	G1	0	0	Absent
	G2	0	0	Absent
	G3	0	0	Absent
	G4	0	0	Absent
D 120	G1	0	0	Absent
	G2	0	0	Absent
	G3	0	0	Absent
	G4	1.0 × 10	0	*Bacillus* sp.

## 11 Discussion

The anatomical protocol using PSS, EA and formaldehyde in different proportions, in addition to 30% SCAS tanks for preservation, was efficient throughout the process, with little release of fat from the specimens. This is similar to that described in EA tanks for fixation (without formaldehyde and PSS) and preservation in 30% SCAS (Zero et al. 2020), especially when using a longer period of fixation in the solution (90 and 120 days), compared to shorter periods (30 and 60 days), probably due to the fat‐solvent action of EA.

The use of EA as a fixative was also effective in cat cadavers for 90 days (Fração et al. 2019), in canine cadavers for 120 days (Rocha et al. 2018), in the analysis of pig viscera through the use of bladder and small intestine of fresh pigs (jejunum), obtained from slaughterhouses, subjected to fixation in EA 99.8% and preserved in 30% SCAS, for 7, 14 and 21 days (Guaraná et al. 2021), both used for surgical training, in addition to human cadavers for 6 months to 1 year, with tissues remaining similar to those of fresh cadavers (Goyri‐O'neill et al. 2013). In this present study, the cadavers did not show contamination or tissue deterioration; the solution maintained the colour, muscle softness and mobility of the joints after 30 days of preservation in 30% SCAS, in a similar way to what occurred in human cadavers injected with EA and glycerine and preserved in thymol (Hammer et al. 2011, 2012) and Modified Thiel solution (Hammer et al. 2014).

The 30% SCAS was evaluated for 5 years for the preservation of cadavers used in anatomy classes, which were previously subjected to fixation with formaldehyde. The specimens remained firm, without visual contamination or unpleasant odour, and without loss of softness or colour (Oliveira 2014). This solution proved to be effective in preserving canine cadavers fixed with EA for 120 days (Rocha et al. 2018) and in cat cadavers fixed and preserved with the same anatomical technique for 120 days for surgical training (Zero et al. 2020). When nitrite and sodium nitrate are added to sodium chloride, pickling salt is formed, which has already been used on cat cadavers fixed together with EA and refrigerated for 90 days (Fração et al. 2019), as well as on cadavers of cats and dogs kept refrigerated and vacuumed for 7 days (Queiroz, Rodrigues, Cardozo, Costa, Soares, et al. 2022; Queiroz, Rodrigues, Cardozo, Costa, Rocha, et al. 2022; Queiroz et al. 2022), 120 days (Queiroz, Rodrigues, Cardozo, Costa, Rocha, et al. 2022) and 120 days (Ferreira et al. 2021) respectively in these species. The salt concentration was always greater than 20%, as recommended for the preservation of corpses (Friker et al. 2007). The PSS used in research of long bones' BM demonstrated that the colour was markable after 30 days, with a more vivid appearance than a fresh corpse, and similar to the colour of a fresh carcass in 60 days (Rocha et al. 2022). The same could be observed in this study using PSS and formaldehyde. The better colour could be explained using the pickling salt solution, widely used in food industries for the maintenance of the meat's colour (Iamarino et al. 2015).

The MRF of skin and jejunum (at the end of preservation in 30% SCAS) ranged from 70.84 N to 158.17 N (average of 123.2 N) and from 14.13 N to 18.76 N (average of 16.4 N), respectively. In canine cadavers subjected to fixation with EA and kept in 30% SCAS, the MRF in these tissues ranged from 106.7 N to 177.5 N (average of 142.1 N) and from 12.9 N to 27.6 N (average of 20.2 N) respectively, demonstrating a discrete effect on biomechanics, even with the use of formaldehyde, which may suggest a good tissue effect caused by the use of 30% SCAS. Vacuum packaging and refrigeration, after fixation of cadavers of dogs with EA and PSS, also showed little variation in biomechanical values in skin MRF between 179.9 N and 139.7 N (average of 156.2 N) for 7 days (del Ponti et al. 2021) and between 125.9 N and 95.78 N for 4 months (Ferreira et al. 2021).

Biomechanical analysis of the skin of cat cadavers subjected to fixation with EA and PSS kept in refrigeration demonstrated MRF at 90 days ranging from 234.7 N to 108.17 N, values higher than those reported in this research because cats presented greater cutaneous elasticity in relation to dogs (Fração et al. 2019). Our values are also much lower when compared to samples from cats chemically prepared in tanks with EA (up to 90 days) and kept in 30% SCAS (up to 120 days); in these animals, the skin MRF ranged from 156.8 N to 217.3 N (average 180.9 N) and the jejunum MRF ranged from 21.9 N to 25.09 N (average 23.6 N) (Zero et al. 2020). With refrigeration, after fixation with EA and PSS, cat cadavers presented MRF of the skin and jejunum ranging from 237.5 N to 337.8 N (average of 270.8 N) and from 19.6 N to 27.6 N (average of 22.95 N), respectively. There was little biomechanical variation between the fresh samples and the anatomical technique described in this research.

Only in one of the 20 analysed moments (group 4, D120) was there bacterial growth (*Bacillus* sp.), however in low quantities (1 × 10 CFU/mL), unlike the contamination frequently reported in the oral cavity, chest surface and anus of canine cadavers subjected to fixation with 30% SCAS stored in the refrigerator at 4°C after 1 month of practical laboratory classes (Nam et al. 2020). This low contamination is similar to that reported in cat cadavers prepared with EA and PSS and kept refrigerated for 1 week, under vacuum, never exceeding bacterial counts of 10^4^ CFU/mL for total aerobes and total anaerobes (*Micrococcus* sp., *Bacillus* sp., *Klebsiella* sp., *Staphylococcus* sp.) (Queiroz, Rodrigues, Cardozo, Costa, Soares, et al. 2022). The longer refrigeration and vacuum time (4 months) of cats chemically prepared with this same technique represented even less microbiological contamination, not exceeding 8 × 10^2^ CFU/mL in total aerobes and 5 × 10^2^ CFU/mL in total anaerobes (*Bacillus* sp., *Staphylococcus* sp. and yeast). In the present study, immersion of cadavers in 30% SCAS for up to 4 months probably considerably reduced contamination due to the hyperosmolarity of the medium (Gauthier et al. 1987; Munro et al. 1989), similar to what occurs in the Dead Sea or in storage vats for corpses previously subjected to fixation by formaldehyde (Nissenbaum 1975; Oliveira 2014).

The fixative and preserving solution containing 23% sodium chloride with Nitrite, 30% ethanol, 20% Pluriol E 400, Oil of oregano and 23.9% tap water can also be used as an alternative to formaldehyde for embalming canine cadavers, although there may be microbiological contamination by *Enterococcus* spp., *Staphylococcus* spp., *Micrococcus* spp., *Bacillus* spp. and *Clostridium perfringens* after 24 weeks in tanks with temperatures varying between 4° and 6° (Janczyk et al. 2011). This solution, similar to that applied in the present study, provided good maintenance of muscle colour, texture and joint movements, especially when a higher percentage of EA was used in the proportion of 30% and even when only formaldehyde was applied, as all cadavers had previously received PSS to fixation, which acted as a protector of natural tissue colour, regardless of the combination of fixing agents used. There was no tissue yellowing as when using antioxidants (Nam et al. 2020).

## 12 Conclusions

The proposed new anatomical technique composed of PSS, 30% EA and 70% formaldehyde provides embalmed cadavers with good morphological and biomechanical appearance similar to the fresh cadaver, in addition to excellent microbiological control for use in anatomy classes and, eventually, surgery training.

## Conflicts of Interest

The authors declare no conflicts of interest.

## Data Availability

The data that support the findings of this study are available on request from the corresponding author. The data are not publicly available due to privacy or ethical restrictions.
